# Replanting of first‐cycle oil palm results in a second wave of biodiversity loss

**DOI:** 10.1002/ece3.5218

**Published:** 2019-05-07

**Authors:** Adham Ashton‐Butt, Simon Willcock, Dedi Purnomo, Suhardi, Anak A. K. Aryawan, Resti Wahyuningsih, Mohammad Naim, Guy M. Poppy, Jean‐Pierre Caliman, Kelvin S.‐H. Peh, Jake L. Snaddon

**Affiliations:** ^1^ Department of Biological and Marine Sciences University of Hull Hull UK; ^2^ School of Biological Sciences University of Southampton Southampton UK; ^3^ School of Natural Sciences Bangor University Gwynedd UK; ^4^ SMART Research Institute (SMARTRI) Riau Indonesia; ^5^ Conservation Science Group Department of Zoology University of Cambridge Cambridge UK; ^6^ School of Geography and Environmental Science University of Southampton Southampton UK

**Keywords:** agriculture, belowground, ecosystem function, invertebrate, Macrofauna, soil, sustainability

## Abstract

1. Conversion of forest to oil palm plantations results in a significant loss of biodiversity. Despite this, first‐cycle oil palm plantations can sustain relatively high biodiversity compared to other crops. However, the long‐term effects of oil palm agriculture on flora and fauna are unknown. Oil palm has a 25‐year commercial lifespan before it must be replanted, due to reduced productivity and difficulty of harvesting. Loss of the complex vegetation structure of oil palm plantations during the replanting process will likely have impacts on the ecosystem at a local and landscape scale. However, the effect of replanting on biodiversity is poorly understood.
2. Here, we investigate the effects of replanting oil palm on soil macrofauna communities. We assessed ordinal richness, abundance, and community composition of soil macrofauna in first‐ (25‐ to 27‐year‐old) and second‐cycle oil palm (freshly cleared, 1‐year‐old, 3‐year‐old, and 7‐year‐old mature).
3. Macrofauna abundance and richness drastically declined immediately after replanting. Macrofauna richness showed some recovery 7 years after replanting, but was still 19% lower than first‐cycle oil palm. Macrofauna abundance recovered to similar levels to that of first‐cycle oil palm plantations, 1 year after replanting. This was mainly due to high ant abundance, possibly due to the increased understory vegetation as herbicides are not used at this age. However, there were subsequent declines in macrofauna abundance 3 and 7 years after replanting, resulting in a 59% drop in macrofauna abundance compared to first‐cycle levels. Furthermore, soil macrofauna community composition in all ages of second‐cycle oil palm was different to first‐cycle plantations, with decomposers suffering particular declines.
4. After considerable biodiversity loss due to forest conversion for oil palm, belowground invertebrate communities suffer a second wave of biodiversity loss due to replanting. This is likely to have serious implications for soil invertebrate diversity and agricultural sustainability in oil palm landscapes, due to the vital ecosystem functions that soil macrofauna provide.

## 1 INTRODUCTION

Oil palm plantations currently cover more than 21 million ha of the tropics (FAO (Food and Agriculture Organization of the United Nations), 2017). Conversion of forests to oil palm has resulted in huge biodiversity losses, especially in Southeast Asia where 85% of palm oil is produced (Koh & Wilcove, 2008; Savilaakso et al., 2014). Large, negative impacts on species richness have been recorded after forest conversion to oil palm in birds; mammals; invertebrates; and fungi (Brühl & Eltz, 2010; Edwards et al., 2014; Fukuda, 2009; Lees et al., 2015; Shuhada, Salim, Nobilly, Zubaid, & Azhar, 2017). However, the long‐term effects of oil palm cultivation on biodiversity are understudied, with the majority of studies focussing on the immediate impacts after forest conversion (Kurz et al., 2016; Savilaakso et al., 2014). Oil palm has a 25‐year commercial lifecycle. As plantations age, yield decreases and palms become difficult to harvest due to their height (Corley & Tinker, 2016). In large‐scale oil palm plantations, replanting usually involves the clear cropping of palms by heavy machinery. This involves pushing over mature palms with a bulldozer or digger and uprooting them. The boles of the felled palms (and sometimes the trunks) are then shredded and distributed on the soil surface, where a leguminous cover crop and the young oil palms are planted (Corley & Tinker, 2016). By 2030, over 13 million ha of first‐cycle oil palm plantations are likely to have been replanted (FAO (Food and Agriculture Organization of the United Nations), 2017).

Although oil palm has much lower biodiversity than rainforest, it is a perennial crop, with a relatively complex vegetation structure and can support a considerable range of species (Foster et al., 2011). Furthermore, agricultural landscapes are becoming increasingly important for biodiversity conservation, in their own right, due to loss of natural habitat (Fahrig et al., 2011; Tscharntke et al., 2012). Current methods of replanting, involving the simultaneous removal of large areas of plantations, could lead to a loss of biological complexity and significantly reduce available habitat for flora and fauna, both locally and at a landscape scale (Luskin & Potts, 2011).

Agricultural intensification and land‐use change have been found to have negative effects on soil biodiversity and ecosystem functioning (Creamer et al., 2015; De Vries et al., 2012; de Vries et al., 2013).As a result, loss of soil biodiversity has been identified as one of the major issues facing soil security and named as a key factor in the six existential global environmental challenges facing humanity (McBratney, Field, & Koch, 2014). The largest genetic and taxonomic diversity of any habitat is found in soil (Lavelle et al., 2006). This biological diversity is important for ecosystem functions such as nutrient retention, carbon cycling, and maintaining plant diversity (de Vries et al., 2013; Wagg, Bender, Widmer, & van der Heijden, 2014) and facilitates many ecosystem services that contribute to human health (Wall, Nielsen, & Six, 2015), for example, provision of food, carbon sequestration, and water retention (Adhikari & Hartemink, 2016). Indeed, enriched levels of soil biota have been found to enhance agricultural sustainability by improving crop yield and nutrient uptake and reduce nitrogen leaching (Bender & van der Heijden, 2015). Furthermore, activity and abundance of soil fauna have been found to positively correlate with other soil characteristics that are beneficial to oil palm yield, although the mechanisms that drive these relationships are not well understood (Tao et al., 2018). In addition, the impact of management on soil biodiversity, within oil palm agriculture, is largely understudied (Bessou et al., 2017).

Here, we investigate how oil palm replanting affects soil macrofauna diversity, abundance, and community composition <1 month, 1 year, 3 years, and 7 years after the replanting event, using a space for time approach. By using a 7‐year chronosequence, we quantify temporal fluctuations in soil macrofauna diversity and abundance, over this period. We predicted that diversity and abundance of soil macrofauna would be negatively affected by the disturbance of oil palm replanting, in addition to change in community composition. However, we expected some recovery of soil macrofauna communities after 7 years, due to the restoration of the understory vegetation and oil palm canopy.

## 2 METHODS

### 2.1 Study area

The study was carried out at an industrial oil palm plantation located in the Siak regency of Riau province, Sumatra, Indonesia (0°55′56″N, 101°11′62″E). The plantation, belonging to PT SMART (Golden Agri‐Resources), was established in 1987 and has been certified by the Roundtable for Sustainable Palm Oil (RSPO). The climate of this region is tropical humid, with a mean temperature of 26.8°C and an average rainfall of 2,400 mm (Tao, Slade, Willis, Caliman, & Snaddon, 2016). The study area was logged in the 1970s, and the resulting logged forest was converted to oil palm from 1985 to 1995. At the regional scale, between 1990 and 2012 tropical forest cover in Riau declined from 63 percent to 22 percent mainly due to oil palm expansion (Ramdani & Hino, 2013). The soil type is ferralitic with gibbsite and kaolinite (Ferric Acrisol according to the FAO classification). In our study site, removal of first‐cycle oil palms for replanting was conducted by large diggers. The trunk was removed from the plantations, and the bole, roots, and dead understory vegetation were shredded and dispersed over the plantation. New palms and a leguminous cover crop (*Mucuna brachteata*) were planted less than a month after old palms were cleared.

### 2.2 Sampling strategy

Sampling took place from April to June 2015. The sample plots were centered on individual palm trees. We selected one tree at random in each of: six different blocks of <1‐month‐old, second‐cycle oil palm; eight different blocks of 1‐year‐old second‐cycle oil palm; nine different blocks of 3‐year‐old replanted oil palm; and ten different blocks of the 7‐year‐old second‐cycle oil palm. We selected two trees at random in six different blocks of first‐cycle oil palm. All palms sampled were at least 50 m apart from each other. The uneven sampling design was due to the availability of blocks from different ages and time constraints regarding the date of replanting. Blocks of oil palm are planted in 150 m by 300 m rectangles, with roads or drainage ditches in between blocks. Oil palm plantations are commonly organized in this way to facilitate access by plantation workers.

Soil and soil surface macrofauna were sampled according to the standard Tropical Biology and Fertility Institute soil monolith method (Bignell, Huising, & Moreira, 2008). This involves removing any ground vegetation (e.g., ferns) within a 25 × 25 cm quadrat and cutting out a block of soil to a depth of 20 cm. Leaf litter was retained and invertebrates collected (Franco et al., 2016). Macrofauna were characterized as fauna visible to the naked eye (Kevan, 1968), and all samples were taken by the same researcher. Worms (Annelida) were placed immediately into formalin, and all other invertebrate taxa were placed in 70% ethanol for identification. The invertebrates were sorted to ordinal level with the exception of: Isoptera to infraorder within the order Blattodea; Formicidae (ants) and Lumbricidae to family level; Chilopoda and Diplopoda to class level; and Hirudinae to subclass. Soil monoliths were taken from both the weeded circle and the windrow of each palm (see Ashton‐Butt et al., 2018; Carron et al., 2015). The weeded circle is a zone around the oil palm trunk, with a radius of approximately 2 m, which is kept clear of vegetation by spraying with herbicides, in order to allow unhindered access to harvesters. The windrow zone is a crescent around the palm, on the outside of the weeded circle that is relatively undisturbed and where pruned fronds are also placed throughout the oil palm lifecycle (Corley & Tinker, 2016). The weeded circle and windrow are known to hold different soil macrofauna abundance and composition (Ashton‐Butt et al., 2018; Carron et al., 2015). Thus, macrofauna were sampled from 45 palms, with two samples taken from each palm (weeded circle and windrow), resulting in a total of 90 soil monoliths.

Ground vegetation surveys were conducted at all 45 palms within a 1 m × 1 m quadrat, placed four times at random within both the weeded circle and windrow. The percentage of ground cover and bare ground was also estimated. The final values used for both vegetation and bare ground covers were the average of estimates made by two observers at each quadrat placed. In addition, plants were identified to species level within each quadrat and the number of individuals recorded.

### 2.3 Statistical analysis

All statistical analyses were performed in R 3.4.4 (R Core Team, 2018). We used linear mixed effects models in R package “*lme4*” (Bates, Maechler, Bolker, & Walker, 2014) to examine the effect of replanting and replanting age on order richness (as the data followed a Gaussian distribution) and generalized linear mixed effects models (GLMM) to examine the effect of replanting on soil macrofauna abundance. We used a negative binomial distribution to fit the GLMM to account for overdispersion and non‐normal distribution of the data (Warton, Lyons, Stoklosa, & Ives, 2016). Replanting age (<1‐month, 1‐year, 3‐year, and 7‐year and first‐cycle oil palm) and sampling zone (windrow or weeded circle) were fitted as categorical fixed effects. Sample plots were fitted as random effects nested within oil palm block, to account for the nested sampling design of first‐cycle plots. Plant species richness and ground cover were also tested as fixed effects in the model building process for both macrofauna abundance and macrofauna order richness. However, after model selection by Akaike information criteria (AICc) (Burnham, Anderson, & Huyvaert, 2011) and assessment of the model fit, they were not included in the final model. Significance of replanting age on macrofauna order richness was explored via best linear unbiased predictions (BLUP) and p‐values computed by Kenward–Rodger approximation (Luke, 2017).

To determine whether replanting affected soil macrofauna community composition, we fitted multivariate generalized linear models to the macrofauna abundance data using R package “*mvabund*” (functions “manyglm” and “anova.manyglm”) (Wang, Naumann, Wright, & Warton, 2012). We used this model‐based method to analyse community composition because, unlike distance‐based methods (e.g., PRIMER), multivariate generalized linear models can account for the confounding mean–variance relationships that often exist in ecological count data by modeling multivariate abundance data with a negative binomial distribution (Warton et al., 2016). Model terms were tested for significance with a likelihood ratio test and a Monte Carlo resampling scheme with 999 iterations; we simultaneously performed tests for univariate (single‐order) responses to treatment, adjusting these univariate *p*‐values to correct for multiple testing, using a step‐down resampling procedure (Wang et al., 2012). A significance level of 0.05 was used.

A model‐based approach was used to visualize change in soil macrofauna community composition. A pure latent variable model was fitted using Bayesian Markov Chain Monte Carlo (MCMC) estimation in the R package “*boral*” (Hui, 2016). Default model parameters were used. Posterior latent variable medians from the model were plotted in an ordination in order to visualize potential clustering of first‐ and second‐cycle oil palm sites based on soil macrofauna composition, where the first two axes represent the two most important axes of macrofauna variation (Hui, 2016).

Separate linear mixed effects models with plant species richness and vegetation cover as response variables were fitted with replanting age (<1‐month, 1‐year, 3‐year, and 7‐year and first‐cycle oil palm) and sampling zone (windrow or weeded circle) fitted as categorical fixed effects to examine the effect of replanting age on plant species richness and plant cover.

## 3 RESULTS

### 3.1 Soil macrofauna

**Figure 1:**
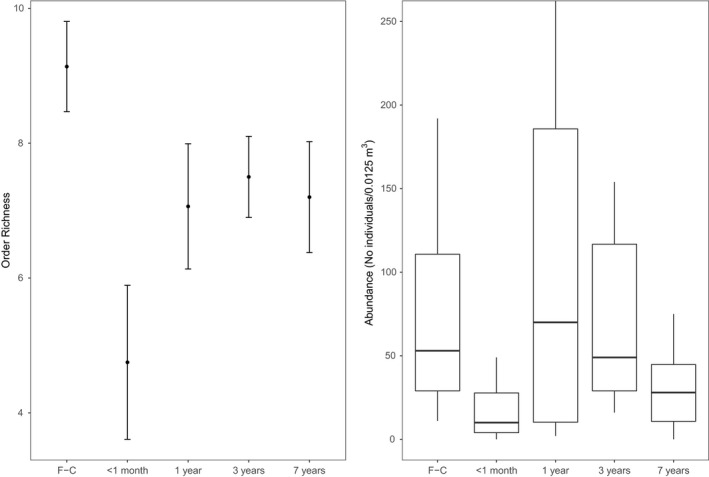
Soil macrofauna ordinal richness and abundance in first‐cycle (F‐C) oil palm and second‐cycle oil palm ages: <1 month, 1 year, 3 years, and 7 years. Box and whisker plots are presented for abundance due to the non‐normal distribution of the data, with horizontal lines representing 25%, 50%, and 75% quantiles and whiskers representing range within 1.5× of the lower or upper quantile. Data outside this range are plotted as individual points. Mean and *SE* were plotted for order richness as data were distributed normally, filled circles indicate means, and bars indicate standard errors

We sampled formi6679 soil arthropods from 37 different orders and taxonomic groups. Formicidae (ants) made up over 50% of all macrofauna (3817 individuals). Other common groups were Lumbricidae (673), Isoptera (304), Aranae (264), Blattodea (222), Dermaptera (221), Isopoda (219), Chilopoda (209), Coleoptera (193), Diplopoda (191), and Diplura (102). These groups contributed to 39% of all macrofauna, and with ants totaled to over 95% of all individuals sampled. Soil macrofauna ordinal richness was lower in all replanting ages than in first‐cycle oil palm (Figure 1, Table 1) although this was marginally statistically significant for 3‐year‐old second‐cycle oil palm (*P *=* *0.083). Ordinal richness was considerable higher in the windrow compared to the weeded circle (Table 1).

**Table 1:** Model outputs of LMMs and GLMM comparing macrofauna order richness, abundance, plant species richness, and vegetation cover between first‐cycle and second‐cycle oil palm ages: <1 month, 1 year, 3 years, and 7 years. First‐cycle oil palm weeded circle is the model intercept, and all other model estimates are compared to this value

Predictors	Macrofauna order richness			(Log) Macrofauna abundance			Plant species richness			Vegetation cover		
	Estimates	CI	*p*	Estimates	CI	*p*	Estimates	CI	*p*	Estimates	CI	*p*
First‐cycle	7.22	5.84 to 8.60	**<0.001**	3.62	3.05 to 4.18	**<0.001**	3.05	2.18 to 3.92	**<0.001**	41.20	28.21 to 54.19	**<0.001**
<1 month	4.38	−6.45 to −2.31	**<0.001**	−1.52	−2.40 to −0.63	**0.001**	−3.06	−4.36 to −1.76	**<0.001**	−56.07	−76.56 to −35.58	**<0.001**
1 year	−2.07	−3.97 to −0.17	**0.033**	−0.02	−0.83 to 0.79	0.964	4.48	3.33 to 5.63	**<0.001**	23.62	5.27 to 41.98	**0.012**
3 years	−1.63	−3.47 to 0.21	0.083	−0.03	−0.80 to 0.73	0.930	−0.12	−1.27 to 1.03	0.836	5.23	−13.12 to 23.59	0.576
7 years	−1.93	−3.72 to −0.14	**0.035**	−0.90	−1.65 to −0.15	**0.019**	0.88	−0.27 to 2.03	0.135	−7.80	−26.15 to 10.56	0.405
Windrow	3.82	2.70 to 4.93	**<0.001**	1.25	0.86 to 1.65	**<0.001**	0.01	−0.76 to 0.79	0.974	23.74	14.80 to 32.68	**<0.001**

Soil macrofauna abundance was lower in <1‐month‐old and 7‐year‐old replanting ages compared to first‐cycle oil palm (Figure 1 and Table 1). Abundance of soil macrofauna was similar between first‐cycle, 1‐year‐old, and 3‐year‐old second‐cycle oil palm (Figure 1 and Table 1). The abundance of macrofauna was higher in the windrow than the weeded circle for all age ranges (Table 1).

**Figure 2:**
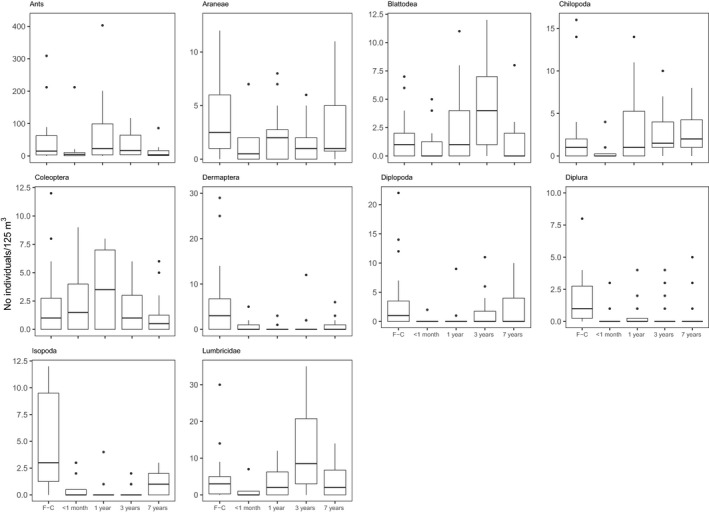
Box and whisker plots of soil macrofauna abundance for the 10 most abundant orders in first‐cycle (F‐G) oil palm and second‐cycle oil palm ages: <1 month, 1 year, 3 years, and 7 years. Horizontal lines represent the 25%, 50%, and 75% quantiles, and whiskers represent the range within 1.5× of the lower or upper quantile. Data outside this range are plotted as individual points

**Figure 3:**
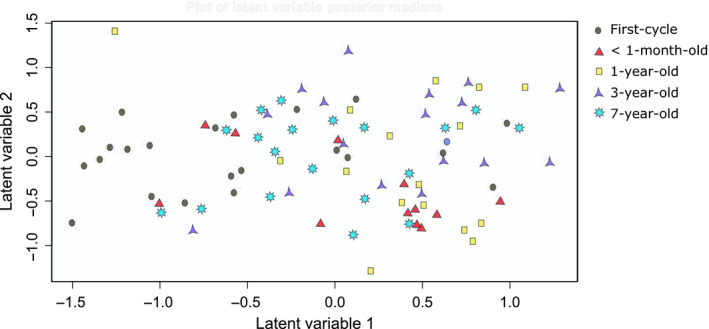
Latent variable model‐based ordination of soil macrofauna composition of first‐cycle and second‐cycle (<1‐month‐old, 1‐year‐old, 3‐year‐old, and 7‐year‐old) oil palm sites

Soil macrofauna order composition changed between first‐ and second‐cycle oil palm sites (LR = 490.4, *P *<* *0.001), and all replanting ages showed statistical difference from first‐cycle oil palm. Of the ten most abundant orders, nine had adjusted univariate *p* values that were significant at the 0.005 level and showed difference in abundance between replanting ages: Formicidae, Blattodea, Chilopoda, Coleoptera, Isopoda, Lumbricidae, Dermaptera, Diplopoda, and Diplura. Only Aranae abundance did not differ between second‐cycle and first‐cycle oil palm. Dermaptera, Diplura, and Isopoda abundance was reduced in all ages of replanting compared to first‐cycle oil palm (Figure 2). The latent variable model‐based ordination showed clear clustering of the first‐cycle sites when compared to the second‐cycle sites (Figure 3). Macrofauna composition of second‐cycle sites of different ages was more similar to each other than first‐cycle sites; however, clustering within age groups was still evident. In addition, soil macrofauna composition was different between the windrow and weeded circle (LR = 181.4, *p *<* *0.001). The abundance of ants (LR = 13.287, *p *<* *0.005), Aranae (LR = 18.6, *p *<* *0.001), Dermaptera (LR = 21.42, *p *<* *0.001), Diplopoda (LR = 14.49, *p *<* *0.001), Diplura (LR = 14.01, *p *<* *0.001), and Isopoda (LR = 19.64, *p *<* *0.001) were all lower in the weeded circle of second‐cycle oil palm when compared to the weeded circle of first‐cycle oil palm. Coleoptera and ants had a higher abundance in the windrow, but not the weeded circle of 1‐year‐old second‐cycle oil palm than in the other replanted ages and first‐cycle oil palm.

### 3.2 Vegetation

**Figure 4:**
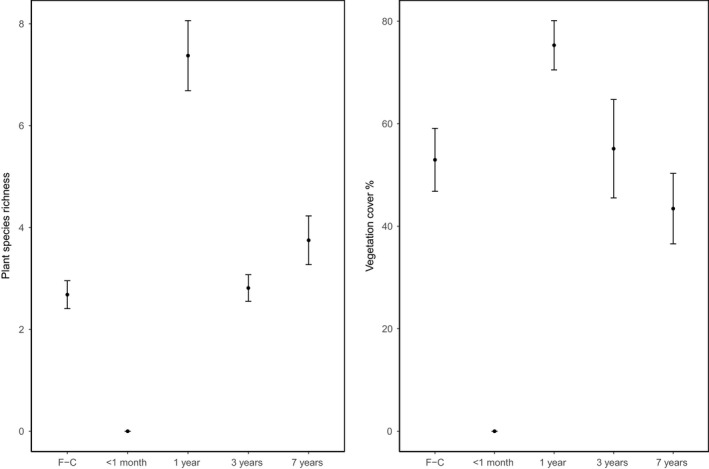
Plant species richness and vegetation cover in first‐cycle (F‐G) oil palm and second‐cycle oil palm ages: <1‐month, 1 year, 3 years, and 7 years. Filled circles indicate means, and bars indicate *SE*

Ground vegetation cover was completely removed after replanting (<1 month); however, cover (model estimate = +22.4%, *p *<* *0.001) and plant richness (model estimate = +4.7, *p *<* *0.001) increased beyond first‐cycle levels 1 year after replanting and then returned to first‐cycle levels 3 years after replanting. There was no difference between vegetation cover or plant richness between first‐cycle, 3‐year, and 7‐year‐old oil palm (Figure 4, Table 1).Vegetation cover was much more extensive in the windrow (model estimate = +25%, *p *<* *0.001) than in the weeded circle, whereas plant richness was the same in both windrow and weeded circle.

## 4 DISCUSSION

### 4.1 Reduction in macrofauna abundance and order richness after replanting

Our study shows that replanting causes a marked decrease in soil macrofauna richness and abundance, in addition to a change in community composition. Worryingly, diversity and abundance of soil macrofauna were still lower and composition was still different, when second‐cycle plantations reached maturity (i.e., 7 years after replanting).

A primary reason for the decline in macrofauna could be the loss of soil organic matter (SOM). SOM is a key food resource for many soil invertebrates (Brussaard, de Ruiter, & Brown, 2007; Brussaard, Pulleman, Ouédraogo, Mando, & Six, 2007). During the replanting process, soil is left completely denuded of vegetation and is disrupted and compacted by heavy machinery. This leaves the soil vulnerable to heavy tropical rains and likely results in large amounts of erosion which removes habitat and nutrients for soil macrofauna (Pimentel & Kounang, 1998). Initial erosion is likely to leave the soil increasingly vulnerable to future erosion by reducing the stability of soil and the capacity for infiltration (Berhe, Harte, Harden, & Torn, 2007; Hamza & Anderson, 2005), further impacting soil macrofauna abundance and diversity during the years after replanting. The subsequent ability of soil macrofauna populations to recover and recolonize may be inhibited by degraded soil. There is also a reduction in inputs of organic matter to soil after replanting, such as rotting vegetation; undergrowth; root matter; and decaying trunks. This reduces food and habitat availability for soil and soil surface macrofauna, compounded by physical disturbance caused by large machinery used to cut down mature oil palms during replanting (Tsiafouli et al., 2015). Large‐bodied and relatively long‐lived soil fauna have been shown to be particularly sensitive to disturbance by agriculture (Postma‐Blaauw, de Goede, Bloem, Faber, & Brussaard, 2010; Tsiafouli et al., 2015).

In addition to physical disturbance of the soil medium, there is a large change in microclimate due to the loss of canopy cover and understory vegetation. The removal of palms and undergrowth during replanting exposes soil to higher temperatures than in mature plantations (Luskin & Potts, 2011). Hot and dry conditions can be unsuitable for many soil macroinvertebrates that are suited to cool, moist conditions, and tropical invertebrates can be particularly sensitive to rises in temperature (Fayle et al., 2010; Kingsolver et al., 2011; Robinet & Roques, 2010). There are few studies on the impacts of disturbance and land‐use change on soil fauna in oil palm plantations. However, species richness and abundance of litter‐dwelling ants substantially decrease after forest conversion to oil palm, likely due to a change in microclimate, increase in disturbance, and reduction in habitat complexity (Fayle et al.,^2010^; Foster et al., 2011). We suggest that the disturbance caused by replanting oil palm is similar to that of land‐use change or intensive agricultural practices, as the relatively complex habitat and diverse vegetation structure of mature plantations are removed.

Interestingly, soil macrofauna abundance recovered to first‐cycle levels in 1‐ and 3‐year‐old second‐cycle oil palm, but dropped to 41% of first‐cycle levels when the plantation reached maturity (7 years of age). This temporary recovery of macrofauna abundance could be due to the increase in vegetation richness and cover, 1 year after replanting. Herbicides use is reduced within the first year, leading to a rapid colonization of plant species and, therefore, high availability of food and habitat resources for insects that are tolerant to disturbance events. Ants were found in extremely high abundance in this age class with a relative contribution per sample of over 55% of invertebrate individuals. Some ant taxa, particularly non‐native species, have been found to be very tolerant to disturbance and extreme microclimates and are found in very high abundance in oil palm plantations (Fayle et al., 2010).

### 4.2 Change in soil macrofauna composition after replanting

Macrofauna composition changed between first‐cycle and second‐cycle oil palm ages. Of the 10 most abundant groups, eight (ants, Araneae, Blattodea, Coleoptera, Dermaptera, Diplopoda, Diplura, and Isopoda) were more abundant in first‐cycle oil palm than 7 years after replanting. This reduction of the majority of the most abundant groups in our study likely reflects the habitat degradation caused by replanting. Reduction in these orders after habitat disturbance and degradation has been found in studies in other habitats (Barnes et al., 2014; Parfitt et al., 2010; Tsiafouli et al., 2015). Abundance of some orders, including ants, Blattodea, and Coleoptera, actually increased between 1 and 3 years after replanting, likely due to the increase in plant diversity and cover due to the halting of herbicide usage, but then fell again between 3 and 7 years when plant diversity and cover dropped. This suggests that the reduction of diversity and abundance of soil macrofauna due to replanting could be buffered by using lower levels of herbicides; increased vegetation could also prevent soil degradation and aid regeneration of SOM (Ashton‐Butt et al., 2018) as seen in other crops (Keesstra et al., 2016; Parfitt et al., 2010). We recognize that due to the relatively coarse level of identification of soil macrofauna in this study, more nuanced relationships of diversity and community composition change between oil palm ages may have been missed. Thus, we predict that our findings on the negative impacts of replanting on soil biodiversity are likely conservative. Due to the staggering diversity of soil macrofauna and the poor understanding of tropical soil fauna taxonomy, further identification was out of the scope of this study. However, we did endeavor to include orders such as Diplura, which are often ignored in tropical soil biota studies (Carron et al., 2015; Franco et al., 2016).

Isoptera were found in low abundances in all oil palm ages, similar to findings from the previous studies (Carron et al., 2015; Luke, Fayle, Eggleton, Turner, & Davies, 2014). Isoptera are considered ecosystem engineers in tropical ecosystems, providing ecosystem functions such as decomposition of wood and SOM and thus playing important roles in nutrient cycling (Lavelle, 1997). Isoptera are found in very high abundances in the natural habitat in this region (tropical forest) but require humid conditions to avoid desiccation and soils rich in organic material for colony building and food (Eggleton, 1997; Hassall et al., 2006). Replanting causes a hotter and drier microclimate (Luskin & Potts, 2011) and reduces organic material in soil (Ashton‐Butt et al., 2018); Isopteran abundance, especially of soil feeding species, is likely to be severely impacted in areas with high densities of oil palm plantations, possibly causing local and even regional extinctions of these species.

### 4.3 Influence of oil palm zone on abundance and richness

Macrofauna abundance was 70% lower, and order richness was 35% lower in the weeded circle than in the windrow, according to our model estimates and in agreement with a previous study (Carron et al., 2015). The weeded circle is relatively devoid of vegetation, receives higher levels of chemical fertilizers and herbicides, and is exposed to more disturbance by oil palm workers than the windrow (Carron et al., 2015). This finding highlights the importance of understory vegetation for soil biodiversity in oil palm plantations. Simplified understory in oil palm has been linked with lower above‐ and belowground invertebrate densities and decreased ecosystem functioning (Ashton‐Butt et al., 2018; Spear et al., 2018). An increased understory could provide protection and refuge for soil organisms during and after the replanting event. Vegetation cover and plant species richness were not good predictors of abundance or order richness in our models, however. There may have been an interaction effect of vegetation with replanting age. However, these effects could not be included in our model due to insufficient sample sizes.

### 4.4 Potential impacts on ecosystem functions

Reductions in soil biodiversity and abundance have been found to have negative effects on ecosystem functions and primary productivity such as nutrient retention; litter decomposition; carbon sequestration; SOM formation; and plant diversity (Handa et al., 2014; Lavelle et al., 2006; de Vries et al., 2013; Wagg et al., 2014). It is likely that after oil palm replanting, there will be a synergistic effect from the degradation of soil biodiversity and soil quality, slowing or preventing soil rehabilitation. Loss of soil functionality could have a negative effect on oil palm yield and the future viability of the soil as a medium for growing crops (Brussaard, de Ruiter, & Brown, 2007; Brussaard, Pulleman, Ouédraogo, Mando, & Six, 2007). Of the more abundant macrofauna groups, decomposers were badly affected, with abundance of Diplopoda, Diplura, and Isopoda decreasing substantially after replanting and remaining low when plantations reached maturity. This could have a knock on effect on nutrient cycling, as these decomposers are considered functional keystone groups (Hättenschwiler, Tiunov, & Scheu, 2005; Wall, Bardgett, Behan‐Pelletier, Jones, & Herrick, 2012) that transform the soil habitat by processing large amounts of litter (Heemsbergen et al., 2004; Jean‐Francois & Gillon, 2002) and influence the composition of microbial decomposers and smaller soil fauna (Hättenschwiler et al., 2005). High abundances of these orders have been linked with greater decomposition rates in oil palm plantations (Ashton‐Butt et al., 2018.).

## 5 CONCLUSIONS

Soil macrofauna abundance, ordinal richness, and community composition are adversely affected by replanting of oil palm. This has worrying implications for the conservation of soil biodiversity in areas with large concentrations of oil palm plantations. Furthermore, this loss of soil biodiversity is likely to impact ecosystem functioning, threatening the sustainability of oil palm beyond the first cycle of growth. We found that soil macrofauna temporarily recovered in abundance after replanting, possibly explained by a temporary rise in vegetation diversity, before falling considerably. This demonstrates the importance for future studies to investigate long‐term responses to disturbance events.

## CONFLICT OF INTEREST

None declared.

## AUTHOR CONTRIBUTIONS

A.A.‐B. lead the research and wrote the paper. S.W. helped with the study design and contributed to the writing of the paper. D.P. helped with study design and conducted the vegetation surveys. S., A.A.K.A., and R.W. helped with study design and data collection. G.M.P. helped conceive the study. J.‐P. Caliman helped with study design and contributed to writing the paper. K.S‐H.P. and J.L.S. were principal investigators on the project and were involved in all stages of the study and manuscript preparation.

## Data Availability

We will make data available from the Dryad Digital Repository: https://doi.org/10.5061/dryad.k14t77p
